# Supplementary material for: Comparative multiomics analysis of cell physiological state after culture in a basket bioreactor
Source: Sci Rep. 2022 Nov 23;12:20161. doi: 10.1038/s41598-022-24687-4 (PMC9686226; doi:10.1038/s41598-022-24687-4)
Supplement: Supplementary file 1 — Supplementary Information 1. [file 41598_2022_24687_MOESM1_ESM.zip › raw data/Transcriptomic raw data/5.Differential/3.cluster/hclust/h_subcluster_plots.pdf]

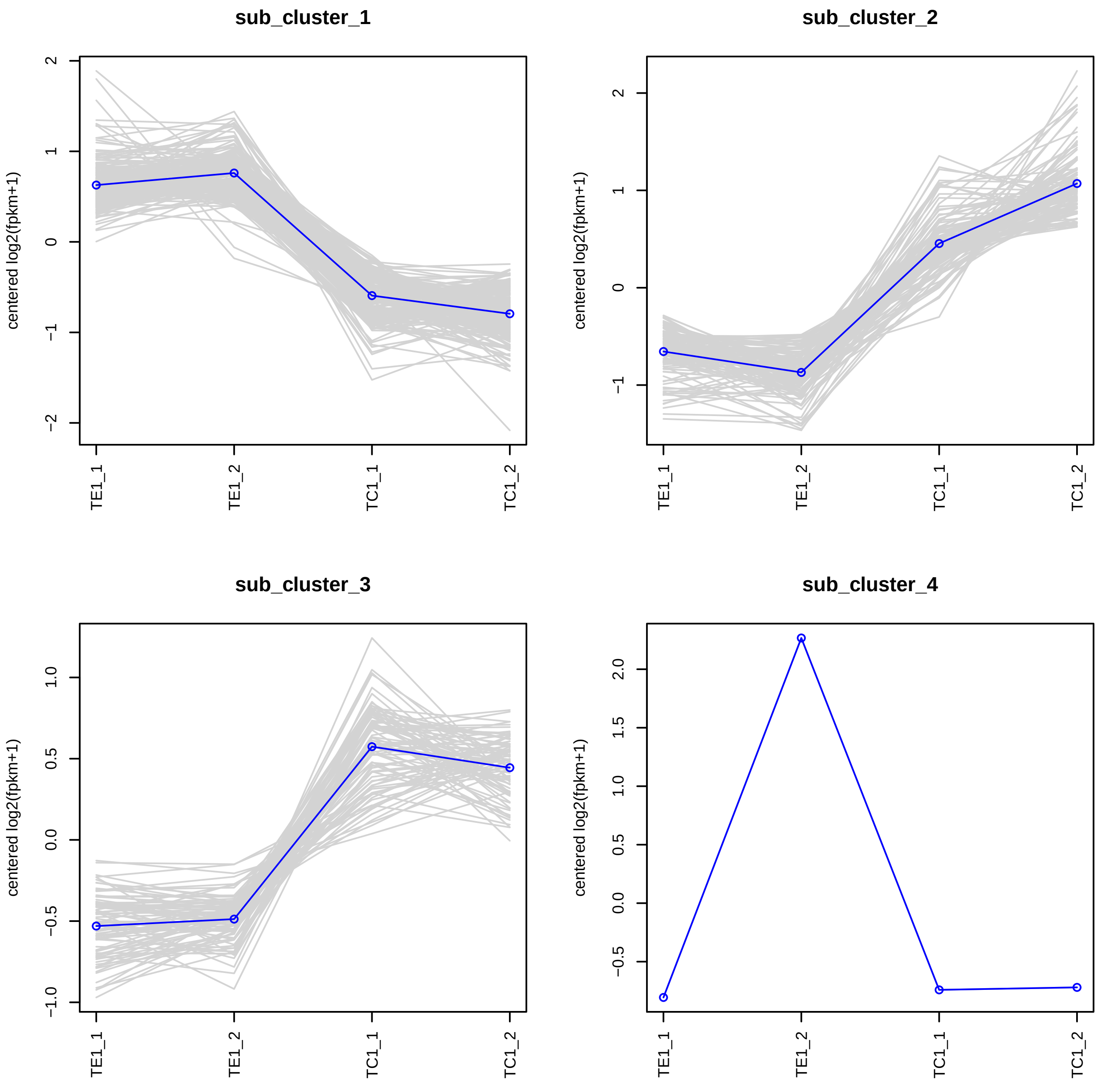
sub_cluster_1
sub_cluster_2
sub_cluster_3
sub_cluster_4
centered log2(fpkm+1)
TE1_1
TE1_2
TC1_1
TC1_2
−2
−1
0
1
2
−1.0
−0.5
0.0
0.5
1.0
1.5
2.0
